# Does information about MIH on dental homepages in Germany offer high quality? A systematic search and analysis

**DOI:** 10.1007/s40368-023-00857-4

**Published:** 2024-02-01

**Authors:** A. Geiken, L. Banz, M. Kock, F. Schwendicke, C. Graetz

**Affiliations:** 1 Clinic of Conservative Dentistry and Periodontology, University of Kiel, Arnold-Heller-Str. 3, Haus B, 24105 Kiel, Germany; 2 Clinic for Conservative Dentistry and Periodontology, University Hospital of Ludwig-Maximilians-University Munich, Goethestr. 70, 80336 Munich, Germany

**Keywords:** MIH, Dental website, Evidence-based dentistry, Internet

## Abstract

**Purpose:**

The internet is increasingly used to seek health information. A dental condition of increasing concern and public interest is molar incisor hypomineralisation (MIH), why we evaluated the information quality of German dentists ‘websites on the topic of MIH.

**Methods:**

A systematic search was performed by two independent investigators using three search engines. The information content of websites on MIH and technical, functional aspects, overall quality, and risk of bias were assessed using validated instruments (LIDA, DISCERN). Practice-related characteristics (practice type, specialization, setting, number and mean age of dentists) were recorded, and associations of these characteristics with websites’ overall quality were explored using multivariable linear regression modelling.

**Results:**

70 sites were included. 52% were multipractices in urban areas (49%). The most common age group was middle-aged individuals (41–50 years). The average number of dentists/practice was 2.5. The majority met more than 50% of the DISCERN and LIDA criteria (90%, 91%). The MIH definition was frequently used (67%), MIH symptoms were described (64%), and 58% mentioned therapies. The prevalence of MIH was mentioned less frequently (48%). MIH example photographs were rarely shown (14%). In multivariable analysis, most practice-related factors were not significant for overall site quality. Only chain practices had slightly higher quality in this regard (2.2; 95% CI of 0.3–4.1).

**Conclusions:**

MIH is mentioned on a large proportion of dentists’ websites. Overall technical, functional, and generic quality was high. Risk of bias is limited. While most websites provided a basic definition of MIH and its symptoms, important information for patients was missing.

## Introduction

Molar incisor hypomineralisation (MIH) is a developmental structural disorder of the tooth structure (Elfrink et al. 2012). According to the definition of the European Academy of Paediatric Dentistry (EAPD), MIH is a hypomineralisation of at least one or more of the first permanent molars with or without the involvement of the incisors (Elfrink et al. 2015). Depending on the severity, teeth with MIH show a creamy-white, brownish-yellowish change in the enamel with or without posteruptive breakdown (Weerheijm et al. 2001). Teeth with MIH are often sensitive to cold and heat, and children often avoid oral hygiene, leading to an increased risk of caries (Leppaniemi et al. 2001). Consequently, MIH patients often show reduced oral health-related quality of life compared to unaffected children (Joshi et al. 2022; Reissenberger et al. 2022).

MIH prevalence is reported very differently in the literature worldwide (Schwendicke et al. 2018), with the mean global prevalence estimated at an average of 878 million people reported to be affected and an expected 17.5 million new cases occurring each year (Schwendicke et al. 2018). The aetiology of MIH has not been sufficiently clarified to date, and various synergistic actions have been discussed (Beentjes et al. 2002; Kühnisch et al. 2014; Lygidakis et al. 2008). In fact, however, there is a great desire among affected children and especially their parents for information about MIH.

For this purpose, the internet represents a medium that is easy to use and accessible at any time. In addition, the majority of the population has access to the internet, and it is therefore understandable (Initiative 21 e.V. 2022) that this is also used to obtain information about health topics (Park et al. 2016).

For example, as early as 2013, 72% of the US population obtained information via the internet (Fox and Duggan 2012). More recent data from Switzerland confirmed this phenomenon and showed even higher usage rates of 91% (Jaks et al. 2019). However, it is not possible to adequately assess the extent to which the information provided comes from possibly non(dental) professionals and whether it can be considered of high quality. There is also a risk of conscious or unconscious misinformation. For example, possible misstatements could mislead patients into resisting established treatment methods or even risk not wanting to take life-defining measures. For example, one hoax is the anticarcinogenic effect of apricot kernels (Cancer Inst. UK. 2015).

However, there is no scientific evidence for this, and it has been demonstrated that excessive consumption of apricot kernels can lead to cyanide poisoning (Vogel et al. 1981). Even more serious is the hypothesis that mumps, rubella and measles vaccines could cause autism (Swire-Thompson and Lazer 2020). This hypothesis has been refuted in several publications, but nevertheless, it continues to be advocated (Taylor et al. 1999). In particular, social media such as Facebook, Instagram, Twitter and YouTube are not evidence-based sources in this regard (Basch et al. 2018, 2019; Graf et al. 2020; Hoffman et al. 2019). Additionally, particularly aggressive behaviour in disseminating nonscientifically supported statements can be observed here (Basch et al. 2019; Kata 2012). Unfortunately, parents cannot be expected to be sufficiently capable of checking existing content for its truthfulness (Eysenbach and Kohler 2002).

Ideally, therefore, the information conveyed should be based on evidence-based (dental) medicine and help patients make informed decisions about therapies. Dental websites should be able to serve as an important source of information. Previous studies evaluating dental websites, including information content about periodontitis or conservative dentistry and options for filling repair, showed a mixed picture (Kanzow et al. 2020; Schwendicke et al. 2017). It would be desirable for dental websites to provide information about MIH, whether that be an explanation of the terminology or treatment options.

To date, no research has investigated whether dental websites can do this.

The aim of this study was therefore to investigate whether German dental websites can provide this information in terms of (1) technical and functional aspects, (2) overall quality and risk of bias, and MIH-specific information. The null hypothesis was that practice-related parameters (age, specialization, location) have no influence on the quality of website information.

## Material and methods

The reporting of this study follows the Preferred Reporting Items for Systematic reviews and Meta-Analyses (PRISMA) and Strengthening the Reporting of Observational studies in Epidemiology (STROBE) guidelines (Cuschieri 2019).

### Sample size calculation

To calculate the required number of sites, a significance level of *α* = 0.05 and an effect size of 0.2 (moderate) were set. The statistical power was estimated to be *β* = 0.80. The calculation was performed using G × Power 3.1.9.2 (University of Düsseldorf, Germany). It was estimated that at least 64 sites would be sufficient (*F* = 2.52; degrees of freedom (df) = 4, expected power 81%). See also the publication by Schwendicke et al. (2017).

### Functional Aspect and generic risk of bias

The LIDA and DISCERN instruments are used to provide users with a systematic assessment of the accessibility, usability, reliability and overall quality of information (Charnock et al. 1999; Minervation 2011). LIDA uses a range of criteria to assess the accessibility, usability (including clarity, consistency, functionality, engagement) and reliability (timeliness, conflicts of interest, content production) of websites. Items are scored on an ordinal scale (0 = never, 1 = occasionally, 2 = most of the time and 3 = always). The points achieved are added to a total score, and the percentage of the maximum possible score is calculated. Scores > 90% represent good results, and scores < 50% represent poor results. In contrast, DISCERN assesses user experience, overall quality and risk of bias of health information for making treatment decisions, reliability (trust in the information and the source of information) and quality of information (treatment alternatives). The rating is on an ordinal scale from 0 (no) to 3 (yes). These instruments have been used in previous studies and are well-established tools (Kanzow et al. 2020; Schwendicke et al. 2017). In addition, a catalogue of criteria was drawn up for the content aspect. Demographic data (size of the city, structure of the practice, age of the dentists, specialization) are shown in Table 1. Regarding MIH, questions included whether the disease was mentioned or defined at all and whether aetiologic/therapeutic aspects were explained. The exact list of questions is given in Table 4.

**Table 1:** Practice-specific parameters of the included websites

Variable and attribute	Value (n (%))
Practice location	
Rural	5 (7%)
City	3 (44%)
Large city	34 (49%)
Practice setting	
Single practice	31 (44%)
Multipractice	36 (52%)
Chain practice	3 (4%)
Age	
Young	3 (4%)
Middle age	39 (56%)
Old	24 (34%)
No information	4 (6%)
Specializations	
Pediatric dentistry	3 (4%)
(German society of pediatric dentistry)	
Any specializations	12 (17%)
No information	55 (79%)

### Search strategy

**Fig. 1:**
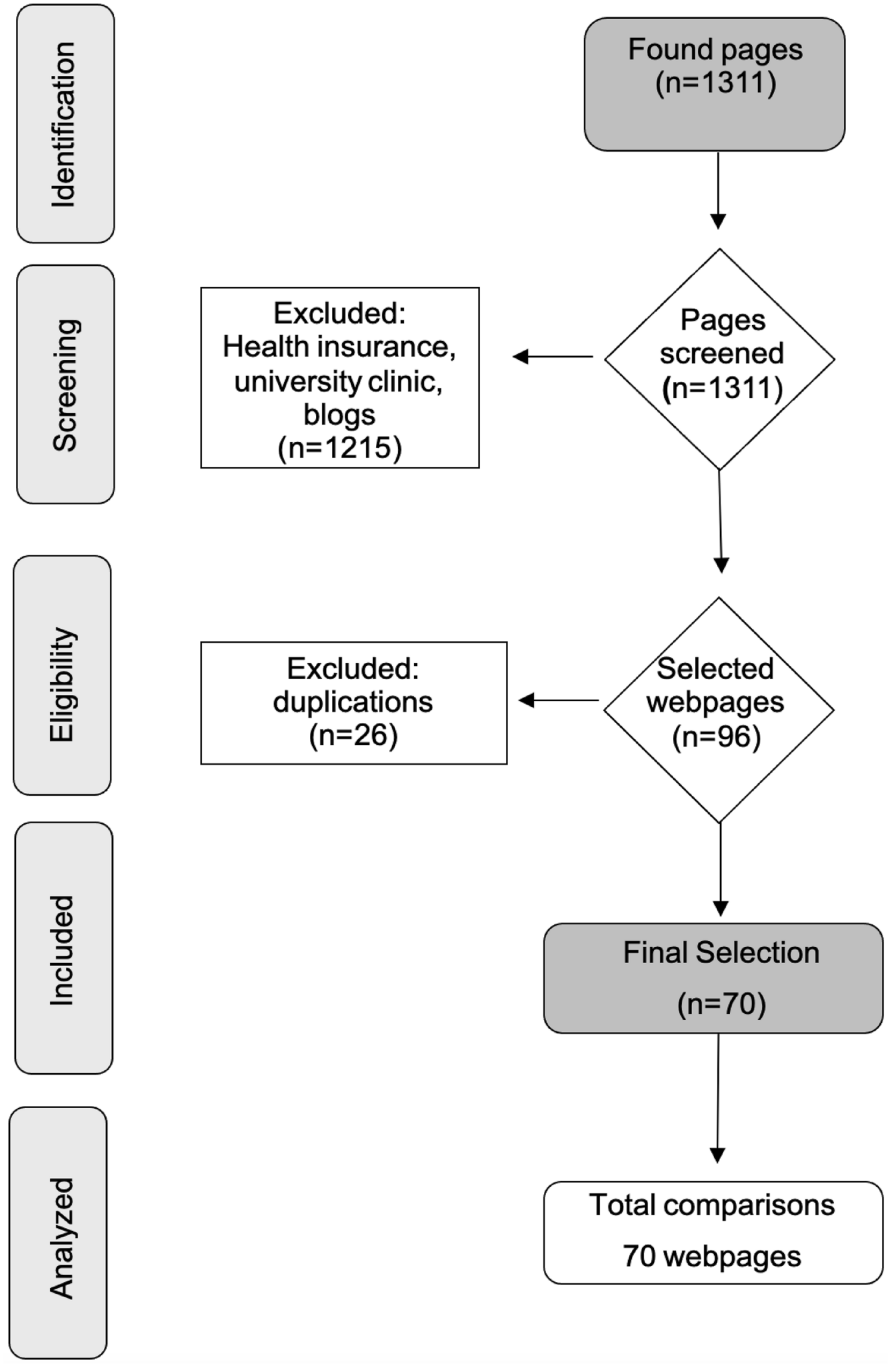
Flowchart of search and selection results

A structured search with a search engine (google.de, bing.de, yahoo.de) was conducted by two calibrated independent examiners (L.B., M.K.) from 10.09.2021 to 12.09.2021 in Germany. Before the search, cookies and browser history were deleted and the browser settings were changed so that Germany was selected as the location and German as the language. The search terms used were "MIH/cheese teeth/dentist", separated by slashes. All 1311 pages found were noted and checked. The 1215 websites of university clinics, health insurance companies or blogs were excluded. 26 duplicates were also eliminated. Finally, there was a selection of 70 pages, which were analysed (Fig. 1). For intrarater reliability and interrater reliability, 10 websites were evaluated consecutively. The re-evaluation took place at 3-month intervals (first evaluation 10.09.2021 to 12.09.21, second evaluation 11.12.21). The reviewers discussed any discrepancies and obtained the opinion of a third reviewer (A.G.) if necessary.

### Data extraction and analysis

Reviewers independently transferred the obtained data of all included websites into a data extraction form. The following data were extracted: practice name, URL, practice location (rural area ≤ 5000 inhabitants, city > 5000—< 100,000 inhabitants, large city ≥ 100,000 inhabitants), practice situation (single practice, multipractice or chain practice), age (young: younger than 41 years, middle aged: 41 to 50 years, old: older than 50 years, identified from CVs from the websites and averaged for multipractice or chain practices), specialization (no information, any specialization, paediatric dentistry) and information on MIH (0 = no information, 2 = complete information) (Tables 1, 2, 3 and 4).

**Table 2:** Domains regarding technical and functional aspects were assessed using the modified LIDA instrument (version 1.2)

Item	Median (IQR^a^; min-max^b^)
1.1 Accessibility	
Is the information available full text without registration, log-in or subscription	2 (0; 2–2)
1.2 Usability	
Is there a clear statement of who this website is for?	2 (0; 1–2)
Is the level of detail appropriate to their level of knowledge? Is the layout of the main block of information clear and readable?	2 (0; 0–2)
Is the navigation clear and well structured?	2 (0; 1–2)
Can you always tell your current location on the site?	2 (0; 0–2)
Do navigational links have a consistent function?	2 (0; 0–2)
Is the site structure (categories or organization of pages) applied consistently?	2 (0; 0–2)
Does the site provide an effective search function?	0 (0; 0–2)
Can you use the site without third-party plugins?	1 (0; 0–2)
Can the user make an effective judgment of whether the site applies to them?	2 (0; 1–2)
Is the website interactive?	1 (0; 0–2)
Does the website integrate non-textual media?	1 (0; 0–2)
1.3 Reliability	
Can users submit comments on specific content?	0 (0; 0–2)
Is site content updated at an appropriate interval?	2 (0; 0–2)
Is it^c^ clear who runs the site?	2 (0; 1–2)
Is it^c^ clear who pays for the site?	1 (0; 0–1)
Can the information be checked from original sources?	1 (0; 0–2)

a IQR: interquartile range

b min–max: minimum and maximum

c Websites’ content

0 = never, 1 = occasionally, 2 = most of the time and 3 = always

**Table 3:** Domains regarding generic quality and risk of bias were assessed using the modified DISCERN instrument

Item	Median (IQR^a^; min-max^b^)
2.1 Reliability	
Are the aims clear?	2 (0; 1–2)
Is it^c^ clear what sources of information were used to compile the publication?	1 (0; 0–2)
Is it^c^ clear when the information used or reported in the publication was produced?	0 (0; 0–1)
Is it^c^ balanced and unbiased?	2 (0; 0–2)
Does it provide details of additional sources of support and information?	1 (0; 0–2)
Does it^c^ refer to areas of uncertainty?	0 (0; 0–2)
2.2 Quality	
Does it^c^ describe how each treatment works?	2 (0; 0–2)
Does it^c^ describe the benefits of each treatment?	2 (0; 0–2)
Does it describe the risks of each treatment?	0 (0; 0–2)
Does it^c^ describe what would happen if no treatment is used?	0 (0; 0–1)
Does it^c^ describe how the treatment choices affect overall quality of life?	1 (0; 0–2)
Is it^c^ clear that there may be more than one possible treatment choice?	2 (0; 2–2)
Does it^c^ provide support for shared decision making?	1 (0; 0–2)

a IQR: interquartile range

b min–max: minimum and maximum

c Websites’ content

0 = no, 1 = occasionally, 2 = most of the time and 3 = yes

**Table 4:** MIH-specific aspects. Scores between 0 and 2 were used

Item	Median (IQR^a^; min-max^b^)
Is MIH/ molar hypomineralization mentioned?	2 (0; 0–2)
Is the term MIH explained in complete sentences?	2 (0; 0–2)
Is it mentioned that the main etiological factor is unexplained?	2 (0; 0–2)
Is it mentioned that the causes are multifactorial?	2 (0; 0–2)
Is it mentioned that MIH is a mineralization disorder?	2 (0; 0–2)
Is a prevalence mentioned?	0 (0; 0–2)
Is mention made of the problems faced by patients?	2 (0; 0–2)
Is it mentioned that affected individuals have an increased sensation of heat/cold?	2 (0; 0–2)
Is it mentioned that atypical hypersensitivity is prominent?	2 (0; 0–2)
Is it mentioned that affected individuals generally have poorer oral hygiene?	0 (0; 0–2)
Is the increased prevalence of caries mentioned?	2 (0; 0–2)
Is the increased level of suffering mentioned?	2 (0; 0–2)
Is it mentioned that affected individuals often have cooperation problems?	0 (0; 0–2)
Is it mentioned that the teeth have an increased risk of fracture?	2 (0;0–2)
Is it mentioned that clearly circumscribed opacities are seen in mild MIH cases?	0 (0; 0–2)
Is it mentioned that post-eruptive enamel breakdown is seen in moderate MIH?	0 (0; 0–2)
Is it mentioned that severe cases may present with atypical restorations and extractions?	0 (0; 0–0)
Is it mentioned that there are different degrees of severity?	0 (0; 0–2)
Is it mentioned that color change may be observed in mild/medium cases?	2 (0; 0–2)
Is it mentioned that crown shape may change in particularly severe cases?	0 (0; 0–2)
Is an example of therapy given?	2 (0; 0–2)
Is it mentioned that therapy depends on many factors?	0 (0; 0–2)
Is it mentioned that fluoridation/sealing and control is sufficient for mild cases?	2 (0; 0–2)
Is it mentioned that for mild to moderate defects, (temporary) filling therapy is performed?	2 (0; 0–2)
Is it mentioned that a steel crown is used for children in the molar region if the defect is severe?	0 (0; 0–2)
Does it mention that extraction with orthodontic gap closure may be required for very severe defects?	0 (0; 0–2)
Is it mentioned that affected individuals should be moved back to a more closely meshed system if possible?	2 (0; 0–2)
Is a prognosis given for the teeth?	0 (0; 0–2)
Are other very similar mineralization disorders/shape changes mentioned?	0 (0; 0–2)
Are there example images of MIH or other mineralization disorders?	0 (0; 0–2)

a IQR: interquartile range

b min–max: minimum and maximum

0 = no information, 2 = complete information

### Statistical analysis

For the descriptive statistical analysis, medians, quartiles and ranges were used, and a quality score (relative percentage, which was the website score for all corresponding items divided by the maximum possible total score) for each domain and overall domains was calculated. Statistical differences in the quality of reporting between domains were tested using Wilcoxon's test. Generalized linear modelling was used to assess the association between practice-related characteristics and overall quality (in %). No interaction terms were used, as this usually requires additional model development, with an increasing risk of alpha-inflation. The statistical analysis was performed with SPSS Statistics for Mac 28.0.0.0 (IBM, Chicago, IL, USA). Statistical significance was set at *p* < 0.05.

## Results

A total of 1311 websites were screened, 1215 were excluded, and 70 webpages were included. The procedure is shown in a flow diagram (Fig. 1). According to the search results an intrarater reliability of 0.946 and an interrater reliability of 0.941 was found for both examiners (L.B., M.K.).

Regarding the practice structure, the majority of the practices were in cities (49%) and were multipractices (52%). Most dentists were middle-aged (56%) (Table 1).

DISCERN and LIDA showed that for technical structure, 99% of the practices scored above 50% of the points to be achieved; and for general information content, 93% scored above 50% of the points (Tables 2 and 3).

The majority of websites lacked information on existing specializations (79%). MIH-specific factors had a high importance (74%) in the mere mention of the term "MIH". Similarly, MIH was frequently defined (67%). Other information about MIH, such as prevalence (48%), patient-specific problems (64%), therapies (58%) and example images of MIH (14%), was rarely mentioned (Table 4). In our multivariable analysis, most practice-related factors were not significantly associated with the overall quality of the website (Table 5). Being a chain practice was associated with minimally increased quality (2.2; 95% CI from 0.3 to 4.1).

**Table 5:** Association between practice-related factors and the overall quality score

Factor	Beta (mean quality score)	95% Confidence interval	*p*-value
Constant	15.3	12.8 to 18.0	< 0.001
Chained practice (ref.: multi- practice)	2.2	0.3 to 4.1	0.053
Single practice (ref.: multi- practice)	0.9	− 0.2 to 1.9	
No information Specialization (ref.: Pediatric dentistry)	− 0.7	− 2.5 to 1.3	0.718
Any Specialization (ref.: Pediatric dentistry)	− 0.8	− 2.9 to 1.2	
Rural (ref.: large city)	0.0	− 1.4 to 1.5	0.127
City (ref.: large city)	− 0.7	− 1.5 to 0.0	
Age of dentists (cont., per year))	− 0.2	− 0.7 to 0.3	0.445
Number of dentists (cont.)	0.0	− 0.2 to 0.3	0.536

rural (≤ 5.000), city (> 5000- < 100.000), 3—large city (≥ 100.000)

## Discussion

MIH is considered a disease with a particularly high care burden. Affected children show a reduced quality of life and increased dental therapy needs (Bandeira Lopes et al. 2021) and is becoming increasingly prominent for patients and parents and usually have a great desire to understand the disease but also options to manage it. Therefore, nothing is more obvious than for patients and parents to use the internet for this purpose. The internet is accessible at all times and usually holds information about diseases and health issues free of charge (Miniwatts Marketing Group 2022). It is therefore an important medium for helping patients make informed decisions and is increasingly used to clarify health issues. Aguirre et al. (2020) showed that internet searches for MIH are increasing worldwide. However, not all internet resources appear to be suitable for this purpose (Eysenbach and Kohler 2002; Schwendicke et al. 2017) and there is a risk of false statements when information is obtained from social media (Basch et al. 2018, 2019; Hutchison et al. 2020; Wang et al. 2019). Professionals such as dentists, however, should provide objective, reliable and accurate information on health aspects in the spirit of shared decision-making.

The evidence to date on the information quality of dental homepages is limited. Studies have been published on periodontology, restorative dentistry, craniomandibular dysfunction and orthodontics (Akan and Dindaroglu 2020; Kanzow et al. 2020; Patel and Cobourne 2015; Schwendicke et al. 2017). LIDA and DISCERN, which were also used in this study, are established tools for assessing the quality of technical aspects, generic quality and risk of bias of websites (Aguirre et al. 2017; Akan and Dindaroglu 2020; Kanzow et al. 2020; Patel and Cobourne 2015; Schwendicke et al. 2017). These aspects generally scored high in our study, which is comparable to and confirms previous national studies on German dentists’ websites (Kanzow et al. 2020; Schwendicke et al. 2017). In contrast, there was a clear contrast in the presentation of the dental information content of MIH. Weaknesses were evident here. Although there was usually some information about MIH (mainly on the clinical appearance and the definition of MIH), more detailed information was less frequently available. For example, although more than half of the websites (58%) described treatment recommendations, only 48% provided the prevalence of MIH. A graphic representation of the disease in the form of photographs or the like was used by only 14%. A photographic presentation of MIH may be helpful for parents to distinguish MIH from differential diagnoses such as fluorosis or amelogenesis imperfecta.

A significant difference in website quality in terms of the location of the practice (rural, small town, large city) or the structure of the practice (single, multipractice, chain practice) could not be found in our study.

However, this aspect was also confirmed by previous studies (Kanzow et al. 2020; Schwendicke et al. 2017). Additionally, the practice structure or age of the dentist did not significantly affect the overall quality of the website. Previous research (Schwendicke et al. 2017) showed a better quality of dental information content, in this case on the topic of periodontitis, on websites of specialized dentists. Unfortunately, due to the very low proportion of German dentists specializing in paediatric dentistry, our study was likely underpowered for such analysis.

This study has several limitations. For example, only German websites were considered and not international websites. Thus, the results could possibly have been influenced by national factors (e.g., specialization, insurance system or specifications for content and design of websites by dental associations). The evaluation procedure was performed by two independent investigators (L.B. and M.K.), and their intra- and interrater reliability was high. A last potential weakness was the compilation of the criteria catalogue for MIH-specific aspects. There is currently no catalogue of expectations regarding what information a website should present, which is why a new criteria list and validated it jointly with a specialist group of paediatric dentists and dentists was developed.

## Conclusions

Within the study limitations, it could be shown that the quality of German websites was good, but the information about MIH, when mentioned, only covered a certain basic knowledge. Further information about MIH was hardly given. In this context, the dental profession, as the operator of the websites, should try to provide better information as well as imaging of MIH to counter misinformation.

## Data Availability

Not applicable.

## Abbreviation

MIH: Molar incisor hypomineralisation
